# Has teaching about intellectual disability healthcare in Australian medical schools improved? A 20-year comparison of curricula audits

**DOI:** 10.1186/s12909-020-02235-w

**Published:** 2020-09-21

**Authors:** Julian N. Trollor, Claire Eagleson, Beth Ruffell, Jane Tracy, Jennifer J. Torr, Seeta Durvasula, Teresa Iacono, Rachael C. Cvejic, Nicholas Lennox

**Affiliations:** 1 Department of Developmental Disability Neuropsychiatry (3DN), UNSW Sydney, 34 Botany Street, Sydney, NSW 2052 Australia; 2 Centre for Developmental Disability Health Victoria (CDDHV), Monash Health, 122 Thomas Street, Dandenong, VIC 3175 Australia; 3 Faculty of Medicine, Nursing and Health Sciences, Monash University, Clayton, VIC 3168 Australia; 4 Department of Psychiatry, School of Clinical Sciences, Monash University, Monash Medical Centre, Block P, Level 3 246 Clayton Rd, Clayton, VIC 3168 Australia; 5 Centre for Disability Studies, The University of Sydney School of Medicine, Faculty of Medicine and Health, Level 1, Medical Foundation Building, 92-94 Parramatta Road, Camperdown, NSW 2050 Australia; 6 La Trobe Rural Health School & Living with Disability Research Centre, La Trobe University, 102 Arnold Street, Bendigo, VIC 3550 Australia; 7 Queensland Centre for Intellectual and Developmental Disability (QCIDD), Mater Research Institute (MRI-UQ), The University of Queensland, Level 2 Aubigny Place, Mater Hospitals, South Brisbane, QLD 4101 Australia

**Keywords:** Intellectual disability, Medical training, Medical education, Curriculum, Health inequalities, Audit comparison

## Abstract

**Background:**

People with intellectual disability (ID) have multiple and complex health needs, more frequent healthcare episodes, and experience poorer health outcomes. Research conducted two decades ago showed that medical professionals were lacking in the knowledge and skills required to address the complex needs of this patient group. The aim of the current study was to determine whether Australian undergraduate medical schools that offer ID health education content had changed the amount and nature of such teaching over this period.

**Methods:**

Identical or equivalent questionnaire items were compared across eight Australian medical schools that participated in curricula audits conducted in 1995 (referred to as T1) and 2013/14 (T2). The audits were of the nature of the ID content, methods used to teach it, and who taught it.

**Results:**

There was no significant difference in the number of hours of compulsory ID content offered to medical students at T2 (total = 158.3 h; median = 2.8 h per ID unit) compared with T1 (total = 171 h; median = 2.5 h). At T2 compared with T1, units with ID content taught in the area of general practice had increased (2 units; 3.6% to 7 units; 16.3%), while decreases were seen in paediatrics (22 units; 40.0% to 10 units; 23.3%) and psychiatry (10 units; 18.2% to 4 units; 9.3%). The number of schools using problem- and/or enquiry-based learning rose to six at T2 from one at T1. Inclusive teaching practices (people with ID develop or deliver content) in compulsory/elective units had increased at T2 (10 units; 23.3%) compared with T1 (6 units; 10.9%), but direct clinical contact with people with ID had decreased (29 units; 52.7% to 11 units; 25.6%).

**Conclusions:**

Overall, little progress has been made to address the gaps in ID education for medical students identified from an audit conducted in 1995. Renewal of ID content in medical curricula is indicated as a key element in efforts to improve workforce capacity in this area and reduce barriers to care, with the aim of reversing the poor health outcomes currently seen for this group.

## Background

It has long been recognised that people with intellectual disability (ID) experience high rates of physical and mental health conditions [1, 2] as well as high mortality rates, often from preventable causes [3–5]. People with ID are more frequent users of emergency departments, ambulatory care settings and hospitals than people without ID [6–8], and thus regularly come into contact with medical practitioners. However, people with ID still experience multiple barriers to accessing quality healthcare, which crucially includes poor workforce knowledge and skills. This, in part, has been associated with a paucity of education for health professionals around the needs of people with ID [9–12].

Almost two thirds of Australian general practitioners responding to a survey on the standards of healthcare for people with ID and educational needs reported a lack of adequate training to care for people with ID, and almost all indicated an interest in receiving further education in this area [13]. Another survey of Australian general practitioners examining barriers and solutions to providing high-quality healthcare to people with ID found that over a third of respondents said that they were not confident treating people with ID [14]. Similarly, Australian consultant and trainee psychiatrists rated their training in ID mental health as inadequate and voiced a lack of confidence to meet the needs of people with ID [15, 16]. While Victorian psychiatrists reported improvements in ID mental health training in the decade prior to 2004, they expressed that further improvements were required [17]. Health needs of people with ID vary depending on the influence of social determinants, including access to effective healthcare services, level of social inclusion, poverty, and support for a healthy lifestyle [18]. They also vary by the level of ability and cause of the ID. Thus, health professionals require content-specific knowledge to appropriately support the health of people with ID. For example, people with mild ID have higher rates of mental ill health [19], while those with higher support needs can experience ill health compounded by the effects of multiple disabilities. People with specific syndromes or genetic causes of ID often have specific health needs, which may require proactive screening and monitoring: for example, multi-system surveillance and management for people with tuberous sclerosis [20]. To reduce barriers to access, specific adaptations to practice are often required, such as tailoring communication [21].

Since deinstitutionalisation, there has been substantial progress in the way Australia has articulated its responsibilities in supporting people with ID, including within health systems and services. Australia signed and ratified the United Nations Convention on the Rights of Persons with Disabilities (UNCRPD) [22]. The National Disability Strategy [23] prioritises the universal equipping of health professionals and services for people with disabilities, while the Fifth National Mental Health and Suicide Prevention plan [24] states that people with ID require a coordinated, accessible, and person-centred approach across services. However, in contrast to other groups with identified health inequalities, such as Aboriginal and Torres Strait Islander peoples [25], no whole of government approach has been forthcoming for people with ID. Such initiatives are crucial as they result in comprehensive policy frameworks, service development, research funding, and equipping of the workforce. An example of a flow-on effect is the equipping of future doctors through the Committee of Deans of Australian Medical Schools Indigenous Health Curriculum Framework [26], with all Australian medical schools implementing aspects of the Framework. The integration of clinical and indigenous health content within lectures was reported as having a positive impact on students’ attitudes towards the health needs of Aboriginal and Torres Strait Islander peoples [27].

In recent decades, medical education pedagogy has evolved. Within the Australian undergraduate medical curricula, there has been increased integration of basic science content and clinical experience [28]. More widely, there is greater student-directed learning, such as that incorporated into problem- and enquiry-based learning (PBL; EBL) [29, 30]. There has also been a movement from didactic lectures to teaching methods that incorporate varied media, such as e-learning and high fidelity simulation that encourage critical thinking [31–33]. In Australia, each university develops its own medical program curriculum, which must meet the Standards for Assessment and Accreditation of Primary Medical Programs developed by the Australian Medical Council (AMC) [34]. The accreditation standards do not specifically mention ID. The Australian Curriculum Framework for Junior Doctors [35], which includes some outcomes relating to cognitive disability, then guides graduates during prevocational training. Within medical education, there is a need and expectation of education around vulnerable populations [36]. The need is further highlighted by the lack of contact with people with disabilities experienced by medical students [37]. Such experiences help them to challenge stereotypes and learn ways to communicate successfully with patients with disabilities [38].

There have been few investigations into what and how undergraduate ID content is taught. Studies conducted with overseas participants have shown that ID education across medical schools has been inconsistent [39], and generally provided within paediatric or psychiatry courses [40]. Inclusive teaching (involving people with ID in the development or delivery of ID content [41]) has been shown to foster more positive attitudes and improve students’ skills and confidence when working with people with ID [41, 42], and can assist with the goal of including people with disability in community life [10]. Lennox and Diggens [43] conducted the first major audit of ID content in curricula across Australian medical schools in 1995. Researchers conducted telephone interviews with representatives from 10 medical schools to examine the amount and nature of teaching in the area of ID. Gaps in teaching and wide variability across medical schools were found. The opportunity for an up-to-date comparison to determine if there have been changes in ID education within Australian medical schools was made possible by the specific inclusion of items in a subsequent audit of 12 medical school curricula conducted in 2013/14 that mapped onto this historical audit [37, 44]. Eight universities participated in both audits.

The aim of this curriculum audit comparison was to determine whether calls to improve ID education have been addressed [43, 45, 46] by making a direct comparison of the audit results across these eight universities that were common to both studies to determine if their medical schools had augmented their ID content over 20 years. The secondary aim of this audit was to inform future measures that may be required to ensure all medical graduates receive adequate education around ID health. We predicted that a substantial increase in ID education and greater use of student-directed learning approaches, such as PBL/EBL, would be expected in light of the gaps identified in the first audit, and human rights and policy development in the intervening years [22, 23].

## Methods

### Recruitment and materials

The methodology and results of the audits conducted in 1995 (Time 1 referred to as T1) [43] and 2013/14 (Time 2 referred to as T2) [37, 44] have been published previously. In brief, at T1 a paper-based structured interview was administered by trained researchers via telephone with representatives from each department within 10 Australian medical schools. The interview was developed for this 1995 study and is reported in Lennox and Diggens [43]. Data were collected on the amount and nature of ID education, including number of hours of teaching, teaching methods, whether content was assessed, who taught the content, and whether there were academic appointments in ID within each department. The researchers used a snowball sampling technique, contacting academics known to the researchers or university department/year coordinators within each medical school.

At T2, emails were sent to the Deans of the 20 Australian medical schools that provide AMC accredited medical degrees, inviting them to take part in the audit. Agreement to participate in Phase 1 of the study was obtained for 14 medical schools. The Dean’s representative for each medical school was interviewed regarding the structure of the medical degree course. In Phase 1, ID content was identified in the curricula of 12 schools, with the course coordinators of those schools then participating in Phase 2. The Phase 2 online survey audit included the number of hours of ID content, the practice areas in which it was taught, teaching methods, and the professional backgrounds of educators. The Phase 1 interview and Phase 2 online survey were developed for this 2013/14 study and have been published in full in Trollor et al. [37].

### Comparison of audit results

A direct comparison of the results for variables common to both audits across the eight universities that participated in both the T1 and T2 audits was completed. There was no response to participate in the T2 audit from two medical schools that took part in the T1 audit. Four medical schools that took part in the T2 audit were established after 1995. See [43] for results from all participating schools that taught ID content in 1995, and [37, 44] for results from all participating schools that taught ID content in 2013/14.

Throughout the analysis, *intellectual disability units* (ID units) refer to discrete course components containing some auditable content specific to ID [37]. Descriptive analyses were used to examine the majority of results, while the Mann-Whitney *U* test or Wilcoxon signed-rank test were used to compare the number of ID units and hours of ID content for each school across the two audits. For non-normally distributed data, the median and interquartile range (*IQR*) are reported.

## Results

### Intellectual disability content within the course program

#### Compulsory intellectual disability units

**Fig. 1:**
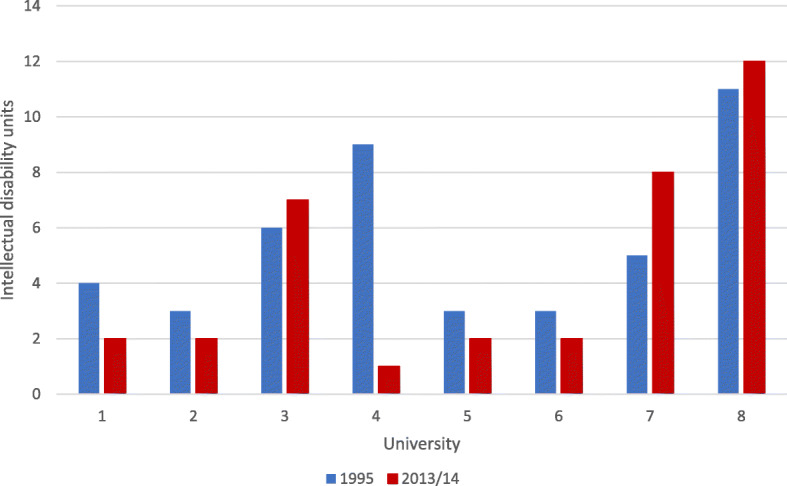
Number of compulsory intellectual disability units per medical school

Across the eight medical schools, there was no significant difference in the number of compulsory ID units (i.e. units all medical students were required to complete) offered at T2 (total = 36; median = 2; *IQR* = 2–7.75) compared with T1 (total = 44; median = 4.5; *IQR* = 3–8.25; *Z* = −.72, *p* = .473). See Fig. 1. for the number of units per medical school. Similarly, there was no significant difference in the number of hours of compulsory ID content taught at T2 (total = 158.3 h, median = 2.8 h per unit; *IQR* = 1.5–5.75;) versus T1 (total = 171 h, median = 2.5 h per unit; *IQR* = 1–3.88; *U* = 724, *Z* = −.66, *p* = .508), nor the number of hours of ID content taught across all compulsory units offered by each school (T2: median = 19.85 h; *IQR* = 4.45–26.75 vs. T1: median = 20.75 h; *IQR* = 8.88–30.50; *Z* = −.17, *p* = .866).

#### Elective intellectual disability units

**Fig. 2:**
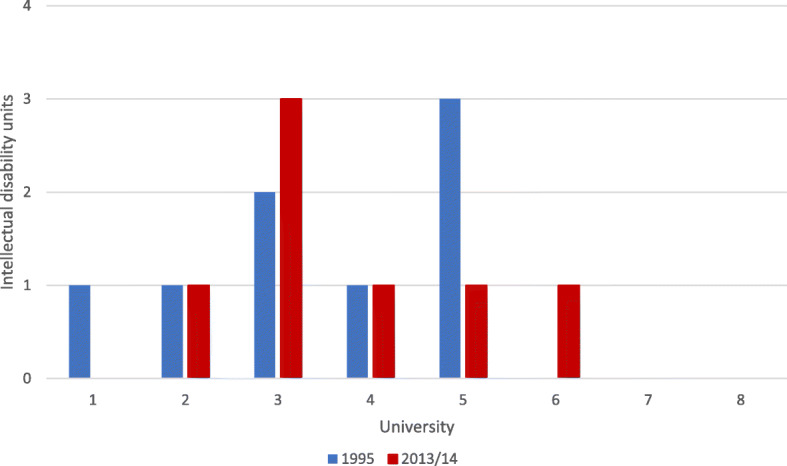
Number of elective intellectual disability units per medical school

Examining ID content in units that only a proportion of medical students completed, five schools offered a total of 7 elective units at T2, compared with a total of 8 elective ID units offered by five schools at T1 (four schools in common at T1 and T2). There was no significant difference in the number of elective ID units offered at T2 (median = 1, *IQR* = 0–1) versus T1 (median = 1, *IQR* = 0–1.75; *Z* = −.38, *p* = .705). Figure 2. displays the number of elective ID units per school. There was no significant difference in elective ID content hours taught at T2 (total = 242 h, median = 3 h per unit; *IQR* = 1–64.5) compared with T1 (total = 173 h, median = 24 h per unit; *IQR* = 12–40; *U* = 10, *Z* = − 1.59, *p* = .112). The number of hours of ID content taught across all elective units offered by each school varied greatly, but was not significantly different at T2 (median = 1 h; *IQR* = 0–3; range = 0–237 h; mode = 0 h) compared with T1 (median = 27 h; *IQR* = 0–48; range = 0–64 h, mode = 0 h; *Z* = −.37, *p* = .715) due to a non-normal distribution, and considerable variability in hours (see range) across a small number of schools, for which the mode was zero hours for both audits. In the T1 audit, it was not known whether 3 units were compulsory or elective.

#### Departments/area of medicine

The number of ID units that were taught within each department/discipline area for each audit are displayed in Table 1, along with the number of schools in which these units were taught. The percentage of ID units that were within the area of paediatrics and psychiatry had decreased at T2 compared with T1, while the percentage of ID units on general practice had increased at T2 compared with T1. Paediatrics was the main department/discipline area in which ID content was taught for both audits.

**Table 1:** Intellectual disability units taught within each department/discipline area of medicine

Department/Discipline area^**a**^	Compulsory units (%; no. schools^b^)		Elective units (%; no. schools)		Total units per discipline (%; no. schools)	
	T1 *n* = 44 units	T2 *n* = 36 units	T1 *n* = 8 units	T2 *n* = 7 units	T1 *n* = 55^c^ units	T2 *n* = 43 units
Paediatrics	19 (43.2%; 7)	8 (22.2%; 6)	3 (37.5%; 2)	2 (28.6%; 2)	22 (40.0%; 8)	10 (23.3%; 6)
General practice	2 (4.5%; 2)	5 (13.9%; 3)	0 (0.0%; 0)	2 (28.6%; 1)	2 (3.6%; 2)	7 (16.3%; 3)
Psychiatry	5 (11.4%; 4)	4 (11.1%; 3)	2 (25.0%; 2)	0 (0.0%; 0)	10 (18.2%; 5)	4 (9.3%; 3)
Other^d^	18 (40.9%; 6)	11 (30.6%; 4)	3 (37.5%; 3)	3 (42.9%; 3)	21 (38.2%; 7)	14 (32.6%; 6)
*Missing* ^e^	–	8 (22.2%; 1)	–	–	–	8 (18.6%; 1)
**Total**	**44 (100%)**	**36 (100%)**	**8 (100%)**	**7 (100%)**	**55 (100%)**	**43 (100%)**

^a^ Department (T1), discipline area (T2). At T2, one or more discipline areas could be reported for each unit; for 8 units that had multiple disciplines areas, only the main discipline area was analysed

^b^ Number of schools across which the units were taught (e.g. 19 compulsory paediatric units were taught across 7 schools at T1)

^c^ Compulsory/elective status was unknown for 3 units at T1 for one school (all psychiatry)

^d^ Other departments at T1 included behavioural sciences, community medicine, geriatric medicine, public health, social and preventative medicine; other discipline areas at T2 included disability, emergency medicine, human development, professional development, sexual health, societal aspects of disability, specialist medicine, women’s health

^e^ Discipline area data was missing for eight compulsory units taught at one school at T2

### How intellectual disability content was taught

#### Methods used to teach intellectual disability content

Table 2 provides a comparison of the number of ID units in which various teaching methods were used at T1 and T2. At T2, there was an increase in the percentage of ID units that incorporated lectures and case studies, and a decrease in ID units with tutorials, seminars/workshops, and other methods (such as role play and clinical demonstrations). The most frequent teaching method used at both T1 and T2 was lectures. At T2, six schools reported using PBL and/or EBL, compared with one school using EBL at T1.

**Table 2:** Intellectual disability units that utilised each teaching method

Teaching method^a^	Compulsory units (%; no. schools^**b**^)		Elective units (%; no. schools)		Total units (%; no. schools)	
	T1 *n* = 44 units	T2 *n* = 36 units	T1 *n* = 8 units	T2 *n* = 7 units	T1 *n* = 55^c^ units	T2 *n* = 43 units
Lectures	24 (54.5%; 8)	26 (72.2%; 6)	2 (25.0%; 1)	2 (28.6%; 2)	27 (49.1%; 8)	28 (65.1%; 7)
Tutorial	8 (18.2%; 4)	5 (13.9%; 3)	2 (25.0%; 2)	2 (28.6%; 1)	11 (20.0%; 6)	7 (16.3%; 3)
Seminar/Workshop	13 (29.5%; 6)	7 (19.4%; 3)	1 (12.5%; 1)	4 (57.1%; 3)	15 (27.3%; 6)	11 (25.6%; 4)
Case study	5 (11.4%; 4)	7 (19.4%; 2)	1 (12.5%; 1)	0 (0.0%; 0)	6 (10.9%; 5)	7 (16.3%; 2)
Other method^d^	8 (18.2%; 4)	2 (5.6%; 2)	1 (12.5%; 1)	3 (42.9%; 3)	9 (16.4%; 4)	5 (11.6%; 4)

^a^ More than one method could be used to teach each ID unit (subsequently no column totals are displayed)

^b^Number of schools across which units were taught

^c^ Compulsory/elective status was missing for 3 units at T1 for one school (lecture, tutorial and seminar/workshop)

^d^ Other methods at T1 included a camp (4-days with 1:1 care of adult with ID), clinical demonstrations, demonstration lecture, interview, role play, and video tapes; and at T2 included clinical assessment, clinical coaching and practicals, clinical day and self-directed learning

Overall there was a decrease in the total number of units (compulsory and elective combined) that involved direct contact with people with ID at T2 (11 units; 25.6% of all ID units; across five schools) compared with T1 (29 units; 52.7% of all ID units; across eight schools). Changes included decreases in the number of compulsory units with direct contact at T2 (7 units; 19.4%; across four schools) compared with T1 (20 units; 45.5%; across seven schools), as well as elective units with direct contact at T2 (4 units; 57.1%; across two schools) compared with T1 (7 units; 87.5%; across four schools). At T1, compulsory/elective status was not known for 3 units from one school, two of which (66.7%) involved direct contact.

#### Assessments

Overall, there was a decrease in the total number of units (compulsory and elective combined) that involved some form of assessment of ID content (e.g. an exam or assignment) at T2 (35 units; 81.4% of all ID units; across eight schools) compared with T1 (53 units; 96.4% of all ID units; across eight schools). At T2, 30 compulsory units (83.3%) across seven schools involved some form of assessment of ID content, a decrease from 42 compulsory units (95.5%) across eight schools at T1. At T2, 5 elective units (71.4%) across three schools had assessments, a decrease from 8 elective units (100%) across five schools at T1. At T1, compulsory/elective status was not known for 3 units for one school, all of which contained some form of assessment of ID content.

#### Who taught intellectual disability content?

A decrease in the reported representation of teaching staff with expertise in ID was observed at T2 compared with T1, with five schools reporting staff with expertise or an interest in ID (or ‘ID champions’) at T2 compared with seven schools reporting that they had an academic appointment in ID and/or staff with expertise/interest in ID at T1. However, information on staff expertise/interest was not available for the remaining three schools at T2.

At T2, compared with T1, there had been an increase in the percentage of ID units that involved medical practitioners, registered nurses, and allied health professionals teaching ID content, but a decrease in ID units that involved psychologists (Table 3). The general role of ‘health professional’ was audited at T1, but not at T2. Compared with T2, there was greater variability of other roles/professions at T1, including representation from family members (of people with ID), an anthropologist, disability professional, and school teacher.

**Table 3:** Intellectual disability units taught by staff from different professional backgrounds

Profession^a^	Compulsory units (%; no. schools^**b**^)		Elective units (%; no. schools)		Total units (%; no. schools)	
	T1 *n* = 44 units	T2 *n* = 36 units	T1 *n* = 8 units	T2 *n* = 7 units	T1 *n* = 55 units	T2 *n* = 43 units
Psychiatrist	7 (15.9%; 4)	7 (19.4%; 4)	0 (0.0%; 0)	2 (28.6%; 1)	7 (12.7%; 4)	9 (20.9%; 4)
Other medical practitioner	22 (50.0%; 6)	23 (63.9%; 6)	6 (75.0%; 4)	6 (85.7%; 4)	28 (50.9%; 8)	29 (67.4%; 7)
Psychologist	11 (25.0%; 6)	3 (8.3%; 2)	0 (0.0%; 0)	2 (28.6%; 2)	11 (20.0%; 6)	5 (11.6%; 3)
Registered nurse	0 (0.0%; 0)	0 (0.0%; 0)	1 (12.5%; 1)	2 (28.6%; 1)	1 (1.8%; 1)	2 (4.7%; 1)
Allied health	4 (9.1%; 4)	5 (13.9%; 3)	1 (12.5%; 1)	5 (71.4%; 3)	5 (9.1%; 4)	10 (23.3%; 5)
Health Professional^c^	9 (20.5%; 5)	–	2 (25.0%; 2)	–	11 (20.0%; 6)	–
Other^d^	10 (22.7%; 6)	1 (2.8%; 1)	2 (25.0%; 2)	0 (0.0%; 0)	12 (21.8%; 6)	1 (2.3%; 1)
*Missing*^ef^	–	8 (22.2%; 1)	–	–	3 (5.5%; 1)	8 (18.6%; 1)

^a^ More than one profession could teach each ID unit (subsequently no column totals are displayed)

^b^Number of schools across which units were taught

^c^ At T1, some professionals only identified as general ‘health professionals’ or ‘medical professionals’; no such data was collected at T2

^d^ Other roles at T1 included anthropologist, behavioural scientist, disability professional, family members (of people with ID), member of Guardianship and Administration Board, person with interest in ID, school teacher, and self-advocacy group member; other roles at T2 included lawyer, person with ID expertise, and social scientist

^e^ Professional background data was missing for eight compulsory units taught in one school at T2

^f^ Compulsory/elective status and profession data was missing for 3 units taught in one school at T1

Overall, there was an increase in the use of inclusive teaching (involving people with ID in the development or delivery of ID content [41]) in ID units at T2 (10 units, 23.3% of all ID units; across seven schools) compared with T1 (6 units; 10.9% of all ID units; across three schools). This change included increases in the number of both compulsory and elective units utilising inclusive teaching across time. For compulsory units, 7 units (19.4%) across five schools were reported to be using inclusive teaching at T2, compared with 5 units (11.4%) across three schools at T1. Similarly, at T2 3 elective units (42.9%) across three schools were reported to be using inclusive teaching, compared with just 1 unit (12.5%) at T1. Compulsory/elective status and inclusive teaching status were missing for 3 units taught in one school at T1.

## Discussion

The current comparison of curriculum audits showed no significant change in the overall amount of ID-specific health education Australian medical students received in 2013/14 compared with 1995 across eight medical schools. While changes were witnessed at specific medical schools, increases in ID teaching in certain schools was countered by decreases in others, resulting in no significant overall change over time. Thus, despite the gaps in ID content and teaching methods identified in the T1 audit [43], our prediction that there would be an increase in ID education over time was not supported. Our second prediction was supported, with more medical schools offering student-directed learning approaches in the form of PBL/EBL. Increasing engagement and variety of clinical teachers, for example medical practitioners, registered nurses, and allied health professionals, appeared to be appropriate responses to the complex health needs of people with ID and need for a multidisciplinary approach to practice [19, 47]. It should be noted that even though the current comparison included only the eight schools that took part in both audits, the number of schools providing compulsory ID education has increased over time, from 10 schools in 1995 [43] to 12 in 2013/14 [37]. However, there was little evidence of broad improvements in the education that future medical professionals receive to help reduce key barriers to health services for people with ID.

An unexpected finding was the decrease in ID education within the areas of paediatrics and psychiatry, two of the most common areas in which ID content was found to be taught in the T1 audit [43]. This finding is of concern as knowledge of the effects of ID on physical and psychosocial health during development is vital for all doctors, as is the ability to recognise and manage mental health issues across the lifespan given the high rates of co-occurring mental ill health in this population group [2, 19, 21]. The increase in ID content taught in the area of general practice was a positive development as people with ID frequently come into contact with health professionals in varied mainstream services [6–8]. Moreover, general practitioners are the primary healthcare providers for this group, but previously a substantial minority had expressed a lack of confidence in caring for people with ID [14], and the majority desired additional training in this practice area [13]. The increase in ID content taught in the area of general practice may provide foundational knowledge and skills for doctors who go on to complete general practice vocational training.

The relative decrease in tutorials and similar methods, and increase in lectures across the audits was unanticipated given the general decline in the use of lectures in undergraduate teaching and tendency toward learning methods incorporating different media that encourage critical thinking [32, 33]. However, the increase in PBL/EBL was in line with the worldwide trend of greater self-directed learning [29, 30]. It was particularly concerning that there has been a decrease in the number of units involving direct clinical contact with people with ID. As the needs of people with ID vary according to the cause and level of ID (as well as other factors), doctors need to tailor their methods of communication, diagnosis, and treatment [1–4, 18, 21]. This tailoring requires responsive, adaptive practice, which is developed through practical experience in varied scenarios. It would appear that only a few students received ID education in clinical settings at T2, with the majority likely having little planned exposure to people with ID. Some students may have had serendipitous contact with people with ID during mainstream service rotations [15]. For curricula set in the late 1980s or early 1990s, it is possible that medical educators with recent experience specialising in ID within institutions may have been more likely to stress the importance of direct clinical contact. There has also been a substantial change in the number and distribution of medical students over this time without a commensurate increase in ID specific clinics (e.g. in mental health due to the mainstream service model in Australia [15]), resulting in a limited number of clinical opportunities with doctors experienced in this area to support students’ learning. Without such contact, students may fail to grasp the need to focus on ways to effectively communicate with patients with ID, and their stereotypes may go unchallenged [38]. Similarly, the positive changes in attitudes seen after personal contact with people with ID, such as increased comfort interacting with people with disability, and viewing the person not the disability, may not be fostered without contact [41, 42].

There was a reasonable representation of educators from different professional backgrounds at the time of both audits. Increases in the ID units taught by four professional groups by T2 would likely have helped students develop awareness of the roles professionals from various disciplines have in the care of people with ID and services that are available to receive referrals. This knowledge is fundamental given the complex needs of many people with ID, with subsequent need for cooperation across the health and disability sectors. In view of the high rates of co-occurring mental ill health and behaviours of concern among people with ID [2], it may be advantageous to have more psychologists providing education around positive behaviour support [48], and highlighting the role of non-pharmacological therapies [49]. The apparent decrease in university staff with expertise in ID (not including inclusive teaching) presents a compounding risk of further erosion of content. In contrast to these concerning trends, the increase in inclusive teaching was encouraging and should be built upon in the future. Including people who have a lived experience of ID is critical to help medical students develop positive attitudes around disability, and develop confidence and skills in communication and adapting assessment and management practices [41, 42]. It also helps include people with disability in community life [10].

The overall results of this audit comparison indicate a lack of response to calls for greater ID content within medical education curricula in Australia [43, 45, 46]. Curriculum development in medical and nursing schools is a key action listed in a draft National Roadmap for Improving the Health of Australians with Intellectual Disability [50], yet there is no timeframe attached to this action. The need to act more swiftly is clear given the lack of action over the past 20 years. While there were improvements in some areas (e.g. a rise in inclusive teaching), the potential impact needs to be considered in the context of declines in other areas (e.g. the decline in clinical contact may have prevented students from practicing skills learnt through inclusive teaching). This lack of progress and considerable variability across medical schools is not entirely unexpected given that each university develops its own medical curriculum and ID health education is not mandatory [34]. We recommend working towards inclusion of a standard specifying minimum graduate capabilities in ID health in the Standards for Assessment and Accreditation of Primary Medical Programs [34], and review of ID content in the junior doctor curriculum framework [35]. Interim pathways could include steps recommended by key experts at the 2018 National Roundtable on the Mental Health of People with Intellectual Disability, such as working with accrediting bodies to gain support, testing a minimum standards curriculum toolkit, and advocating for medical schools to adopt the toolkit to augment course content [51]. A curriculum renewal to enhance ID teaching would have greater impact as part of a whole of government approach, including initiatives such as policy frameworks and equipping the workforce [25].

The results from this comparison require interpretation with some limitations in mind. The T2 audit was not an exact replication of the T1 audit, with some differences in recruitment, data collection, and questionnaire items. However, the comparison was conducted across the same subset of medical schools and for comparable questionnaire items. While the T1 telephone interview allowed participants to provide complete answers and for researchers to clarify ambiguity, the online administration of questionnaires at T2 meant that there were no similar opportunities for clarification and forced-choice questions may have limited the breadth of some responses. There was also no qualitative component in either audit. It would be valuable to determine medical students’ views on the education they receive around ID health and their confidence in working with people with ID. The quantity and method of delivery of ID education may not reflect the quality of the teaching. As part of any future curriculum renewal, consultation should be carried out with students to determine their learning needs, current attributes, and confidence in the area of ID health to i) inform curriculum development and ii) provide a baseline for assessing the impact of a new curriculum. Long-term research (see 17) could also be conducted to assess the effect of changes in curricula content over e.g. a 10-year period on student confidence working with people with ID. Lastly, the most recent audit is now 6 years old, and therefore may not reflect current curriculum content. In future audits, equivalent methodology to that used in the previous audits is warranted, taking into account ways to address the limitations described.

## Conclusions

The current comparison of two audits of ID content in Australian medical school curricula almost 20 years apart showed an overall lack of progress in the amount and nature of medical student ID health education. Action is needed from the Government, education standards committees, universities, and individual educators to ensure that all medical graduates have knowledge of the varied health issues that face people with ID and acquire the skills to make reasonable adaptations to practice. Such knowledge and adaptations are required to reduce barriers to care for people with ID and ensure that they receive the same level of healthcare as the general population, fulfilling the aims of human rights [22] and policy initiatives [23].

## Data Availability

Please contact the first author for data and materials used in this study. The datasets are not publicly available as the authors wish to have a line of communication with researchers interested in this data and be able to explain how to compare variables between datasets.

## Abbreviations

AMC: Australian Medical Council
EBL: Enquiry-based learning
IQR: Interquartile range
PBL: Problem-based learning
UNCRPD: United Nations Convention on the Rights of Persons with Disabilities
